# *In Vitro* Efficacy of Ebselen and BAY 11-7082 Against *Naegleria fowleri*

**DOI:** 10.3389/fmicb.2018.00414

**Published:** 2018-03-06

**Authors:** Anjan Debnath, Andrew T. Nelson, Angélica Silva-Olivares, Mineko Shibayama, Dionicio Siegel, James H. McKerrow

**Affiliations:** ^1^Center for Discovery and Innovation in Parasitic Diseases, Skaggs School of Pharmacy and Pharmaceutical Sciences, University of California, San Diego, La Jolla, CA, United States; ^2^Skaggs School of Pharmacy and Pharmaceutical Sciences, University of California, San Diego, La Jolla, CA, United States; ^3^Department of Infectomics and Molecular Pathogenesis, Centro de Investigación y de Estudios Avanzados del Instituto Politécnico Nacional, Mexico City, Mexico

**Keywords:** parasite, free-living ameba, *Naegleria*, drug, chemotherapy, ebselen, BAY 11-7082, BAY 11-7085

## Abstract

Primary amebic meningoencephalitis (PAM) is a fatal infection caused by the free-living ameba *Naegleria fowleri*, popularly known as the “brain-eating ameba.” The drugs of choice in treating PAM are the antifungal amphotericin B and an antileishmanial miltefosine, but these are not FDA-approved for this indication and use of amphotericin B is associated with severe adverse effects. Moreover, very few patients treated with the combination therapy have survived PAM. Therefore, development of efficient drugs is a critical unmet need to avert future deaths of children. Since *N. fowleri* causes extensive inflammation in the brain it is important to select compounds that can enter brain to kill ameba. In this study, we identified two central nervous system (CNS) active compounds, ebselen and BAY 11-7082 as amebicidal with EC_50_ of 6.2 and 1.6 μM, respectively. The closely related BAY 11-7085 was also found active against *N. fowleri* with EC_50_ similar to BAY 11-7082. We synthesized a soluble ebselen analog, which had amebicidal activity similar to ebselen. Transmission electron microscopy of *N. fowleri* trophozoites incubated for 48 h with EC_50_ concentration of ebselen showed alteration in the cytoplasmic membrane, loss of the nuclear membrane, and appearance of electron-dense granules. Incubation of *N. fowleri* trophozoites with EC_50_ concentrations of BAY 11-7082 and BAY 11-7085 for 48 h showed the presence of large lipid droplets in the cytoplasm, disruption of cytoplasmic and nuclear membranes and appearance of several vesicles and chromatin residues. Blood-brain barrier permeable amebicidal compounds have potential as new drug leads for *Naegleria* infection.

## Introduction

*Naegleria fowleri* has been identified as the cause of primary amebic meningoencephalitis (PAM) in more than 16 countries (Visvesvara and Stehr-Green, 1990). It has been listed by the National Institute of Allergy and Infectious Diseases (NIAID) as a category B priority pathogen. Until 2012, 310 cases had been reported globally with a fatality rate of more than 95% (Gautam et al., 2012). According to the Centers for Disease Control and Prevention (CDC), 143 cases of PAM were reported in the US from 1962–2016 (http://www.cdc.gov/parasites/naegleria/illness.html) (Johnson et al., 2016). While infections were mostly reported from southern-tier states of the US, it is likely that infection with *Naegleria* is underreported because states differ in their capacity to identify, investigate, or report cases (Yoder et al., 2010). Out of the 143 reported cases in the US, 139 have been fatal. PAM occurs disproportionally among children <13 years of age (Yoder et al., 2010). PAM results from water containing *N. fowleri* entering the nasal cavity (De Jonckheere, 2011; Shakoor et al., 2011; Yoder et al., 2012; Centers for Disease Control and Prevention., 2013b), followed by migration of the amebas to the brain. Within the brain, *N. fowleri* causes extensive inflammation, hemorrhage, and necrosis. The time from initial exposure to onset of illness is usually 5–7 days but may be as early as 24 h, leading to death in 3–7 days (Visvesvara et al., 2007).

Optimum treatment for PAM has not been well defined. Amphotericin B remains a cornerstone of therapy for PAM but is not FDA-approved for this indication. Treatment with amphotericin B requires high dosage and its use is frequently associated with renal toxicity, anemia, chills, fever, nausea, vomiting, and headache (McCurdy et al., 1968; Proffitt et al., 1991; Visvesvara, 2010). Moreover, worldwide, no more than a dozen persons with PAM have been treated successfully with amphotericin B alone or in combination with other drugs (Apley et al., 1970; Anderson and Jamieson, 1972; Lawande et al., 1979; Seidel et al., 1982; Brown, 1991; Poungvarin and Jariya, 1991; Loschiavo et al., 1993; Wang et al., 1993; Singh et al., 1998; Jain et al., 2002; Schuster and Visvesvara, 2004; Vargas-Zepeda et al., 2005). Recently, an antileishmanial, miltefosine, has shown some promise in combination with other drugs and a patient was successfully treated (Centers for Disease Control and Prevention., 2013a). However, a second patient, though treated with miltefosine, suffered permanent brain damage. Since effective treatment of PAM requires drugs to cross the blood-brain barrier, identification of blood-brain barrier penetrating anti-PAM leads that can be used as a basis to develop drugs to treat *Naegleria* infection is a critical unmet need to prevent future deaths of children and young adults.

In this study, we selected two blood-brain barrier permeable compounds, ebselen and BAY 11-7082 (Imai et al., 2001; Jayakumar et al., 2014), for testing their activity against *N. fowleri*. Ebselen was reported to be a potent inhibitor of cysteine protease (Nikawa et al., 1994) and could be an antivirulence agent for *Clostridium difficile* infection, affecting cysteine protease activity in the autoprocessing of the toxin B virulence factor (Bender et al., 2015). BAY 11-7082 is a phenyl vinyl sulfone-related compound and phenyl vinyl sulfone compounds are irreversible inhibitors of cysteine proteases (Scheidt et al., 1998; Juliana et al., 2010). Since several studies suggested a possible role of cysteine protease in the pathogenesis of *N. fowleri* (Aldape et al., 1994; Cervantes-Sandoval et al., 2008; Lee et al., 2014; Vyas et al., 2015) and reports from other studies showed that cysteine protease inhibitors representing different chemical scaffold types were effective in halting parasite replication without toxicity to the host (Renslo and McKerrow, 2006), we hypothesized that ebselen and BAY 11-7082 might also inhibit the growth of *N. fowleri*. We showed that both ebselen and BAY 11-7082 inhibited trophozoite growth *in vitro* and demonstrated killing activity as documented by transmission electron microscopy. Because of their efficacy against *N. fowleri* trophozoites, we also synthesized an analog of ebselen and tested this analog and another closely related BAY compound, BAY 11-7085, and showed their activity against trophozoites.

## Materials and methods

### Chemicals and reagents

White, solid bottom tissue culture treated 96-well microplates were purchased from E&K Scientific (Santa Clara, CA). CellTiter-Glo Luminescent Cell Viability Assay was purchased from Promega (Madison, WI); dimethyl sulfoxide (DMSO) and amphotericin B were purchased from Sigma-Aldrich (St. Louis, MO); ebselen (2-phenyl-1,2-benzoisoselenazol-3(2H)-one), BAY 11-7082 or (E)-3-[(4-methylphenyl)-sulfonyl]-2-propenenitrile and BAY 11-7085 or (2E)-3-[[4-(1,1-dimethylethyl)phenyl]sulfonyl]-2-propenenitrile were purchased from Enzo Life Sciences (Farmingdale, NY).

### Compound synthesis and analysis

**Figure 1:**
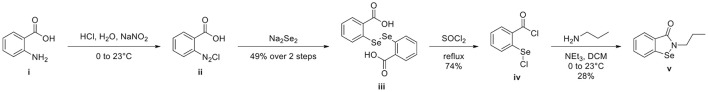
Synthesis of 2-propylbenzo[*d*][1,2]selenazol-3(2H)one **v** from anthranilic acid **i**. 2-propylbenzo[*d*][1,2]selenazol-3(2H)one **v** was prepared in 4 steps from inexpensive, commercially available anthranilic acid **i**. Anthranilic acid **i** was diazotized to afford diazonium salt **ii**, which was treated *in situ* with sodium diselenide, yielding 2,2′-diselanediyldibenzoic acid **iii**. Refluxing **iii** in thionyl chloride gave 2-(chlorocarbonyl)phenyl hypochloroselenoite **iv**. The synthesis concluded by treating **iv** with 1-aminopropane, which, after addition-elimination and substitution, yielded 2-propylbenzo[*d*][1,2]selenazol-3(2H)one **v**.

Ebselen analog 2-propylbenzo[*d*][1,2]selenazol-3(2H)one **v** was synthesized using the steps described in Figure 1. A round bottom flask equipped with stir bar was charged with anthranilic acid **i** (3.00 g, 21.9 mmol, 0.86 eq), deionized water (30 mL), and concentrated hydrochloric acid (4.5 mL). Stirring was initiated, affording a straw-colored solution. An addition funnel was attached. The reaction vessel was submerged in an ice-water bath and aged for 15 min before adding an aqueous solution of sodium nitrite (1.80 g, 26.1 mmol, 1.03 eq in 26 mL deionized water) dropwise by addition funnel. After addition was complete, the reaction was aged 30 min before adding a mixture of sodium diselenide. Sodium diselenide was prepared under a nitrogen atmosphere in a dry round bottom flask equipped with stir bar and reflux condenser. The reaction vessel was charged with selenium powder (2.00 g, 25.3 mmol, 1 eq), sodium metal (0.600 g, 26.1 mmol, 1.03 eq), naphthalene (0.500 g, 3.90 mmol, 0.154 eq), and dry tetrahydrofuran (THF) (50 mL). The reaction vessel was submerged in an oil bath and the black mixture was refluxed for 6 h before cooling to ambient temperature. Stirring was arrested and the supernatant THF was removed via syringe. Dry methanol (0.5 mL) was added dropwise, followed by deionized water (30 mL). Solid sodium hydroxide (1.00 g, 25.3 mmol, 1 eq) was added at once and, after dissolution, the mixture of sodium diselenide was added dropwise to the preformed diazonium salt **ii**. The reaction was allowed to warm to ambient temperature and stirred overnight. Concentrated hydrochloric acid was added dropwise and the precipitate was collected by filtration. The red clay like substance was recrystallized from methanol to afford 4.32 g of 2,2′-diselanediyldibenzoic acid **iii** (49%). Spectroscopic data were in agreement with those previously reported (Selvakumar et al., 2011).

A dry round bottom flask under nitrogen atmosphere equipped with stir bar and reflux condenser was charged with 2,2′-diselanediyldibenzoic acid **iii** (1.00 g, 2.50 mmol, 1 eq) and thionyl chloride (5 mL, 68.9 mmol, 27.6 eq). The reaction vessel was submerged in an oil bath and refluxed for 3 h. The reaction was allowed to cool to ambient temperature. Excess thionyl chloride was removed via rotavap. Hot hexane was added to the black residue and the orange supernatant was decanted away. Upon standing, the orange solution formed spindly, orange crystals 0.94 g (74%) of 2-(chlorocarbonyl)phenyl hypochloroselenoite **iv**. Spectroscopic data were in agreement with those previously reported (Kamigata et al., 1986).

A dry round bottom flask under nitrogen atmosphere was charged with 1-aminopropane (0.082 mL, 1 mmol, 1 eq), dry dichloromethane (DCM) (1 mL, ¼ total volume), and triethylamine (0.249 mL, 2 mmol, 2 eq). Stirring was initiated and the reaction vessel was cooled in an ice-water bath for 15 min before adding a solution of 2-(chlorocarbonyl)phenyl hypochloroselenoite **iv** (0.279 g, 1.1 mmol, 1.1 eq) dissolved in dry DCM (3 mL, ¾ total volume) dropwise via syringe. The reaction was allowed to warm to ambient temperature and stirred overnight. The reaction was diluted with deionized water (20 mL) and DCM (20 mL). The layers were partitioned and separated, keeping the organic layer. The aqueous layer was extracted with DCM (20 mL × 2). Combined organics were dried (sodium sulfate), filtered, and concentrated to afford an orange-brown solid. The crude material was purified by flash column chromatography, eluting with hexanes/ethyl acetate (70/30). Unmixed fractions were concentrated to afford 66 mg of 2-propylbenzo[*d*][1,2]selenazol-3(2H)one **v** as a creamy orange solid (28%). Spectroscopic data were in agreement with those previously reported (Bhabak and Mugesh, 2009).

### Maintenance of *N. fowleri*

Trophozoites of pathogenic *N. fowleri* strain KUL were axenically cultured in Nelson's medium supplemented with 10% FBS at 37°C (Debnath et al., 2017). Trophozoites were counted using a particle counter (Beckman Coulter, Fullerton, CA). All the experiments were performed using trophozoites and cells harvested during the logarithmic phase of growth.

### *In vitro* studies of ebselen, ebselen analog, BAY 11-7082, BAY 11-7085, and miltefosine against *N. fowleri* trophozoites

The compounds were screened and reassayed for EC_50_ determination against *N. fowleri* trophozoites using a final 8-point concentration range. 2.5 μL of 5 mM stock compounds in 100% DMSO were diluted with 17.5 μL sterile water to yield 625 μM working concentration of compounds. A three-fold serial dilution was then performed yielding a concentration range 625 μM-0.25 μM. From this dilution plate, 4 μL were transferred into the 96-well screen plates followed by addition of 96 μL of trophozoites (10,000 amebas per well) to yield a final 8-point concentration range spanning 25–0.01 μM in final 0.5% DMSO (Debnath et al., 2012). Miltefosine was tested at a concentration range of 200 μM-1.56 μM. The assays were performed in triplicate and assay plates were incubated for 48 h at 37°C. At the end of incubation, the assay plates were equilibrated to room temperature for 30 min, 50 μL of CellTiter-Glo Luminescent Cell Viability Assay (Promega) were added in each well of the 96-well plates. The plates were then placed on an orbital shaker at room temperature for 10 min to induce cell lysis. After lysis, the plates were again equilibrated at room temperature for 10 min to stabilize luminescent signal. The resulting ATP bioluminescence of the trophozoites was measured at room temperature using an EnVision Multilabel Reader (PerkinElmer, Waltham, MA). Negative controls in the screen plates contained 0.5% DMSO and positive controls contained 50 μM amphotericin B (Sigma-Aldrich).

In parallel, to determine the effect of ebselen, BAY 11-7082 and BAY 11-7085 on the growth of *N. fowleri*, 10^4^ amebae were incubated at a concentration range of 50–0.39 μM of ebselen, BAY 11-7082 and BAY 11-7085 for 48 h at 37°C. Control trophozoites were incubated with 0.5% DMSO. Cell numbers were calculated by hemocytometer at the end of incubation. The percentage of viable trophozoites due to treatment at different concentrations of compound was determined by the standard trypan blue exclusion method. Cells stained blue were considered non-viable.

### Data analysis and statistics

Standard assay metrics such as Z′ were calculated for each plate. Percent inhibition relative to maximum and minimum reference signal controls was calculated using the formula:

% Inhibition = [(mean of Maximum Signal Reference Control–Experimental Value)/(mean of Maximum Signal Reference Control–mean of Minimum Signal Reference Control)] × 100

Visualization and statistical analysis of compound screening data were performed using GraphPad Prism software 5.0.

### Transmission electron microscopy

For ultrastructural analysis, 2 × 10^6^ *N. fowleri* trophozoites were incubated with 0.5% DMSO, 6.2 μM of ebselen, 1.6 μM of BAY 11-7082 and 2.3 μM of BAY 11-7085 for 24 and 48 h and then fixed with a solution of modified Karnovsky's fixative (2.5% glutaraldehyde and 2% paraformaldehyde in 0.1 M sodium phosphate, pH 7.2). Samples were post-fixed with 1% (w/v) osmium tetroxide, dehydrated with ethanol and propylene oxide and embedded in epoxy resin. Thin sections (50–60 nm) were contrast stained with 2% uranyl acetate followed by Sato's lead stain for 1 min and thin sections were examined under a Tecnai G2 Spirit BioTWIN transmission electron microscope (TEM) equipped with an Eagle 4k HS digital camera (FEI, Hilsboro, OR).

## Results

### Effect of ebselen, ebselen analog, BAY 11-7082, BAY 11-7085, and miltefosine against *N. fowleri in vitro*

**Figure 2:**
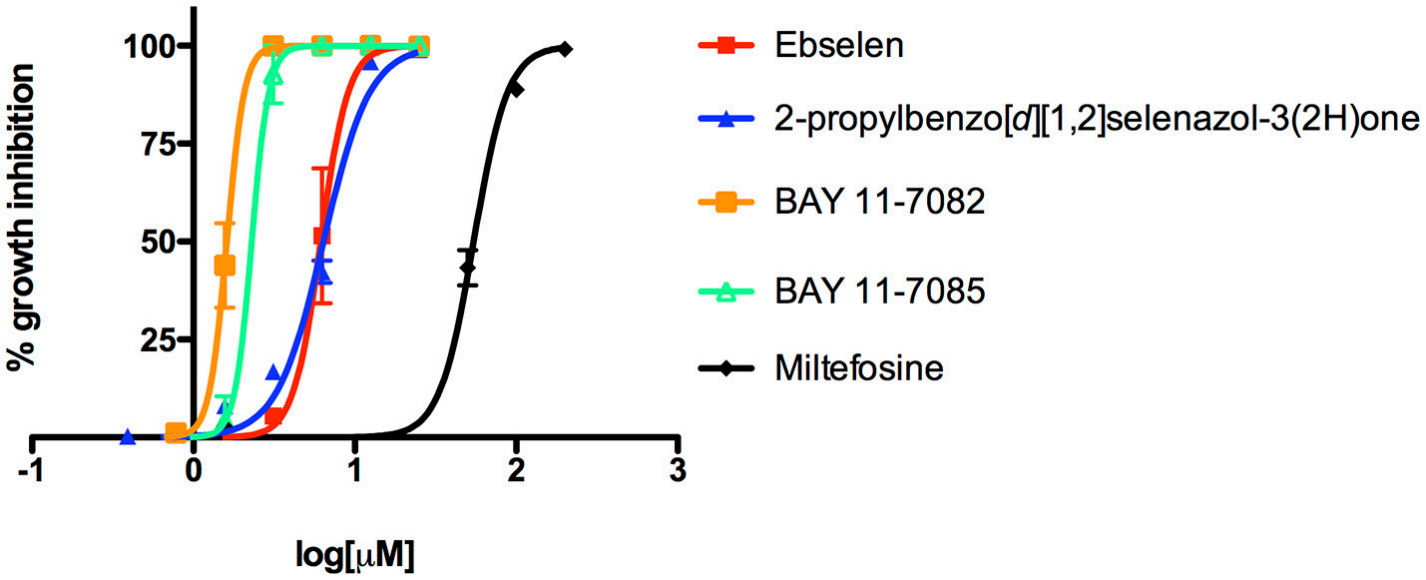
Activity of ebselen, 2-propylbenzo[*d*][1,2]selenazol-3(2H)one, BAY 11-7082, BAY 11-7085, and miltefosine against *N. fowleri*. Different concentrations of compounds were tested for activity (EC_50_) against *N. fowleri* trophozoites. The data represent the mean ± SD.

We adapted a luciferase-based assay, earlier developed with non-pathogenic *N. gruberi* (Debnath et al., 2012), to test the activity of ebselen, ebselen analog, BAY 11-7082, and BAY 11-7085 against *N. fowleri*. Ebselen inhibited *N. fowleri* growth at 12.5 μM (100% growth inhibition) whereas BAY 11-7082 and BAY 11-7085 showed 100% growth inhibition at 3.12 μM. The assay provided a Z′ of 0.92. Based on this ATP bioluminescence-based growth inhibition study, the EC_50_ of ebselen, BAY 11-7082, BAY 11-7085, and miltefosine, defined as that concentration of compound necessary to reduce the culture density to 50% of that of a vehicle-treated culture was approximately 6.2, 1.6, 2.3, and 54.5, respectively (Figure 2, Table 1). The EC_50_ values of ebselen, BAY 11-7082 and BAY 11-7085, determined by trypan blue exclusion method, were 6.1, 1.0, and 1.4 μM, respectively. The data obtained by trypan blue exclusion method were consistent with the EC_50_ values determined by ATP bioluminescence method, though the ATP bioluminescent cell viability assay was more sensitive. In our earlier study (Debnath et al., 2012), we identified BAY 11-7085 as a primary hit against *N. gruberi* and the dose response data reported in this study confirms its activity against pathogenic *N. fowleri*. Since ebselen was found active against *N. fowleri*, we synthesized an ebselen analog with greater solubility. We thought that more soluble analog of ebselen could provide better efficacy at least in cell culture model and tested its activity against *N. fowleri*. Ebselen analog, 2-propylbenzo[*d*][1,2]selenazol-3(2H)one, inhibited the growth of *N. fowleri* with EC_50_ similar to ebselen (EC_50_ = 6.4 μM) (Figure 2, Table 1). While ebselen and its analog were about 8.5-fold more active than the CDC-recommended drug miltefosine (EC_50_ = 54.5 μM), BAY 11-7082 and BAY 11-7085 exhibited 34-fold and 23-fold more potency than miltefosine.

**Table 1:** *In vitro* inhibitory effects of ebselen, 2-propylbenzo[*d*][1,2]selenazol-3(2H)one, BAY 11-7082 and BAY 11-7085.

**Structure**		**EC_50_^a^ (pEC_50_ ± SE) (μM)**
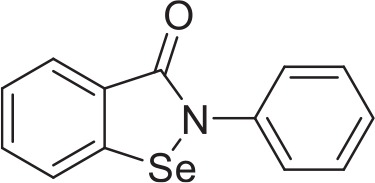	Ebselen	6.2 (5.2 ± 0.02)
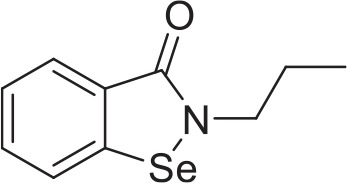	2-propylbenzo[*d*][1,2]selenazol-3(2H)one	6.4 (5.2 ± 0.02)
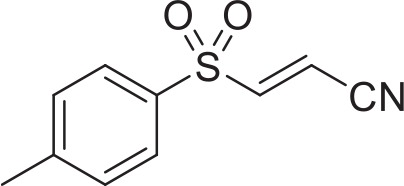	BAY 11-7082	1.6 (5.8 ± 0.02)
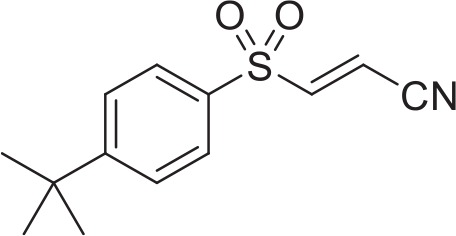	BAY 11-7085	2.3 (5.6 ± 0.04)
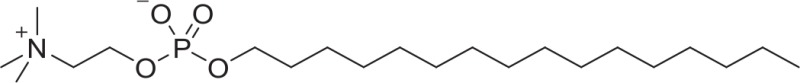		54.5 (4.3 ± 0.01)
	Miltefosine	

a *EC_50_ minimum n = 3*.

### Transmission electron microscopy

**Figure 3:**
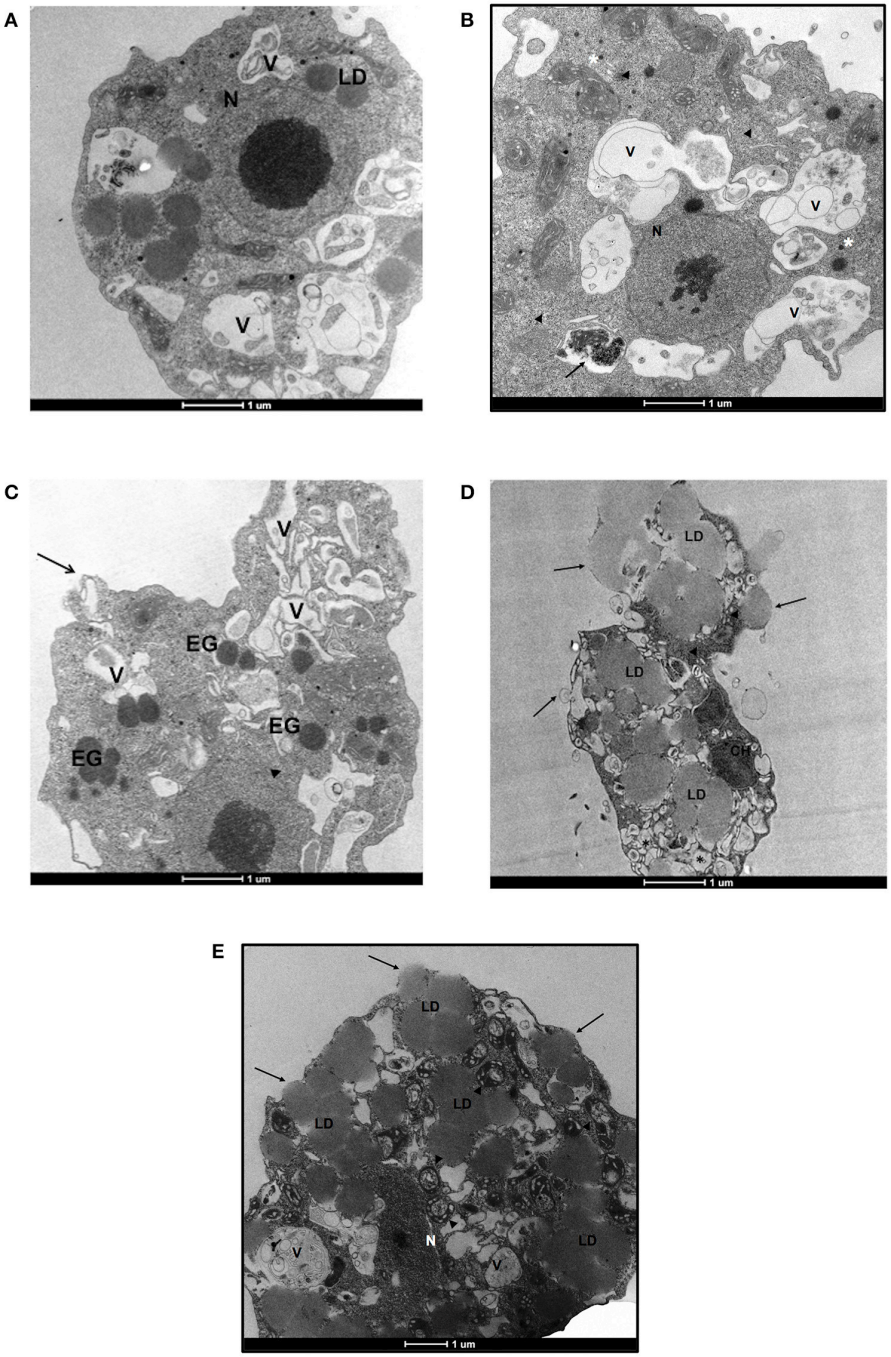
Transmission electron microscopy of *N. fowleri* trophozoites incubated with DMSO, ebselen, BAY 11-7082, and BAY 11-7085. **(A)** Trophozoites treated with 0.5% DMSO. The nucleus and nucleolus appear normal (N). Lipid droplets (LD) and several vacuoles (V) are present. **(B)** Trophozoites treated with 6.2 μM of ebselen for 24 h. The nucleus presents important alterations with loss of chromatin, however, the nuclear membrane appears intact (N). Several mitochondria appear with normal morphology. Some vacuoles are bigger in size with cytoplasmic material, membrane like structures and vesicles of different sizes (V). One vacuole presents mitochondrial and chromatin residues (arrow). Ribosomes (arrow-heads) and electron-dense granules (asterisks) are present in the cytoplasm. **(C)** Trophozoites treated with 6.2 μM of ebselen for 48 h. The cytoplasmic membrane of the amoeba presents a loss in the membrane continuity (arrow). Several vacuoles (V) of different morphology and size are observed. These vacuoles present cytoplasmic content inside. The loss of the nuclear membrane is evident (arrow-heads). The electron-dense granules (EG) appear distributed along the cytoplasm. **(D)** Trophozoites treated with 1.6 μM of BAY 11-7082 for 48 h. Large lipid droplets (LD) are present in the cytoplasm and move the cytoplasmic content toward the periphery of the trophozoite. Several vesicles appear in the trophozoite (asterisks). In the upper side of the ameba, membrane disruption is observed due to the presence of large lipid material (arrows). Chromatin residues (CH) and free ribosomes (arrow-heads) are also seen in the cytoplasm. **(E)** Trophozoites treated with 2.3 μM of BAY 11-7085 for 48 h. Large lipid droplets (LD) are present in the cytoplasm. The nucleus shows alterations, the nuclear membrane disappears and remnants of chromatin are seen (N). The cytoplasmic membrane is also damaged (arrows). The mitochondria appear edematous with loss of mitochondrial crests (arrow-heads). Vacuoles (V) present vesicles and membrane structures that resemble smooth reticulum. Bars, 1 μm.

We also performed a transmission electron microscopy analysis to assess ultrastructural changes in *N. fowleri* induced by EC_50_ concentrations of ebselen, BAY 11-7082 and BAY 11-7085 at 48 h. 0.5% DMSO-treated *N. fowleri* trophozoites displayed normal morphology with several food vacuoles, mitochondria, lipid droplets, and a nucleus containing one large nucleolus (Figure 3A). Forty-eight hours treatment of *N. fowleri* with 6.2 μM of ebselen led to the loss of nuclear membrane and appearance of electron-dense granules. Several vacuoles of different morphology and sizes with cytoplasmic content were also observed. The continuity of the cytoplasmic membrane was also lost (Figure 3C). To determine the effect of ebselen at earlier time point, *N. fowleri* was incubated with 6.2 μM of ebselen for 24 h. At this time point, we observed loss of chromatin but the nuclear membrane appeared intact. Several mitochondria appeared and some vacuoles were bigger in size with membrane like structures. One vacuole presented mitochondrial and chromatin residues. Ribosomes were free in the cytoplasm and electron-dense granules were also present (Figure 3B). At 48 h of exposure of trophozoites to 1.6 μM of BAY 11-7082, disruption of the plasma membrane was evident and large lipid droplets were present throughout the cytoplasm. Large lipid content moved the cytoplasm toward the periphery of the trophozoites. Chromatin residues, free ribosomes and several vesicles were also observed (Figure 3D). Similar ultrastructural changes were also observed when trophozoites were treated with 2.3 μM of BAY 11-7085 for 48 h. BAY 11-7085 induced damage in nucleus and cytoplasmic membrane. The mitochondria appeared edematous with loss of mitochondrial crests. Large lipid droplets appeared in the cytoplasm and vacuoles presented vesicles and membrane structures that resembled smooth reticulum (Figure 3E).

## Discussion

Mortality in PAM exceeds 95% with the current amphotericin B treatment and even if the patient survives there is a possibility that the survivor may experience permanent brain damage or other disabilities. These poor clinical outcomes are due to the extensive brain damage caused by *N. fowleri* and poor penetration of blood-brain barrier by amphotericin B (Nau et al., 2010). Several other compounds have been tested against *N. fowleri* but those compounds had limited activity against the pathogen and could not successfully treat the infection (Duma and Finley, 1976; Goswick and Brenner, 2003). The blood-brain barrier penetration property of compounds was not considered when selecting the agents to test against the pathogen. Therefore, several compounds failed in follow-up studies (Schuster and Visvesvara, 2004). Identification of amebicidal compounds with blood-brain barrier permeability is likely necessary not only for the survival of the patients, but also for their full neurologic recovery.

We evaluated the seleno-organic compound ebselen vs. *N. fowleri* because ebselen has anti-inflammatory properties and has been evaluated in clinical trials for brain hemorrhage (Saito et al., 1998; Yamaguchi et al., 1998). Ebselen is bioavailable, known to be clinically safe, blood-brain barrier permeant, and can achieve brain levels that are 21% of plasma levels (Imai et al., 2001). A phase I safety study using doses of 200–1600 mg was conducted and has led to a phase II clinical trial for noise-induced hearing loss (Lynch and Kil, 2009; Rajguru, 2013). A single oral dose of 100 mg/kg of ebselen in rat yields serum values of 4–5 μM (Salom et al., 2004). An intravenous administration of 1 mg/kg ebselen achieved 12 μg/ml of ebselen concentration in rat plasma (Imai et al., 2001). This concentration is equivalent to about 44 μM of ebselen or more than 7 times the *in vitro* EC_50_ for *N. fowleri*. In a pharmacokinetic study, it was found that the selenium moiety did not become bioavailable and therefore ebselen did not enter the body pool of selenium. Rather it was metabolized and excreted explaining its lack of toxicity (Parnham and Sies, 2000). Both the anti-inflammatory and blood-brain barrier permeability properties make ebselen an attractive compound to test for its activity against *N. fowleri*. The wide range of activities of ebselen is offset by its low solubility. Therefore, we synthesized an analog of ebselen with greater solubility in case it might confer better activity against *N. fowleri in vitro*. The analog did not exhibit a better EC_50_ than ebselen itself. Whether the analog maintains blood-brain barrier permeability will be a subject for future investigation.

BAY 11-7085 and closely related BAY 11-7082 have anti-inflammatory activity (Lee et al., 2012). Treatment with BAY 11-7082 significantly reduced rat brain edema caused by traumatic brain injury (Jayakumar et al., 2014), indicating that it may cross the blood-brain barrier. In another study, treatment with BAY 11-7085 improved the clinical status of rats with pneumococcal meningitis and this beneficial effect was paralleled by a reduction of meningitis-associated CNS complications and meningeal inflammation (Koedel et al., 2000). BAY 11-7082 and BAY 11-7085 have not undergone clinical development, but animal experiments showed limited toxicity and good therapeutic effectiveness (Keller et al., 2006).

We performed an electron microscopy study to identify ultrastructural changes in *N. fowleri* that have been induced by ebselen, BAY 11-7082 and BAY 11-7085. Several reports suggested that mitochondria are potential targets of BAY compounds (Dai et al., 2004; Zanotto-Filho et al., 2010). BAY compounds altered mitochondria of *N. fowleri* at 48 h, while ebselen caused alterations in cytoplasmic vacuolization within 24 h.

In summary, we identified ebselen, BAY 11-7082 and BAY 11-7085 as amebicidals with blood-brain barrier permeability and as potential new drugs for the treatment of PAM.

## Author contributions

AD: Conceptualized the study, performed experiments, analyzed the data, prepared the original draft, reviewed, and edited the manuscript; AN: Performed experiments, contributed to the method section of the manuscript; AS-O: Analyzed the data; MS: Analyzed the data, reviewed, and edited the manuscript; DS: Provided resources and supervised chemical synthesis; JM: Provided resources, reviewed and edited the manuscript.

### Conflict of interest statement

The authors declare that the research was conducted in the absence of any commercial or financial relationships that could be construed as a potential conflict of interest.

## References

[B1] Aldape K. Huizinga H. Bouvier J. McKerrow J. (1994). *Naegleria fowleri*: characterization of a secreted histolytic cysteine protease. Exp. Parasitol. 78, 230–241. 10.1006/expr.1994.1023 8119377

[B2] Anderson K. Jamieson A. (1972). Primary amoebic meningoencephalitis. Lancet 1, 902–903. 10.1016/s0140-6736(72)90772-6 4111856

[B3] Apley J. Clarke S. K. Roome A. P. Sandry S. A. Saygi G. Silk B.. (1970). Primary amoebic meningoencephalitis in Britain. Br. Med. J. 1, 596–599. 5440234 10.1136/bmj.1.5696.596 PMC1699588

[B4] Bender K. O. Garland M. Ferreyra J. A. Hryckowian A. J. Child M. A. Puri A. W.. (2015). A small-molecule antivirulence agent for treating *Clostridium difficile* infection. Sci. Transl. Med. 7:306ra148. 10.1126/scitranslmed.aac9103 26400909 PMC6025901

[B5] Bhabak K. P. Mugesh G. (2009). Amide-based glutathione peroxidase mimics: effect of secondary and tertiary amide substituents on antioxidant activity. Chem. Asian J. 4, 974–983. 10.1002/asia.200800483 19378298

[B6] Brown R. L. (1991). Successful treatment of primary amebic meningoencephalitis. Arch. Intern. Med. 151, 1201–1202. 10.1001/archinte.1991.00400060121021 2043022

[B7] Centers for Disease Control and Prevention (2013a). Investigational drug available directly from CDC for the treatment of infections with free-living amebae. MMWR Morb. Mortal. Wkly. Rep. 62:666. 23965830 PMC4604798

[B8] Centers for Disease Control and Prevention (2013b). Notes from the field: primary amebic meningoencephalitis associated with ritual nasal rinsing–St. Thomas, U.S. Virgin islands, (2012). MMWR Morb. Mortal Wkly. Rep. 62:903. 24226628 PMC4585351

[B9] Cervantes-Sandoval I. Serrano-Luna Jde J. García-Latorre E. Tsutsumi V. Shibayama M. (2008). Characterization of brain inflammation during primary amoebic meningoencephalitis. Parasitol. Int. 57, 307–313. 10.1016/j.parint.2008.01.006 18374627

[B10] Dai Y. Pei X. Y. Rahmani M. Conrad D. H. Dent P. Grant S. (2004). Interruption of the NF-kappaB pathway by Bay 11-7082 promotes UCN-01-mediated mitochondrial dysfunction and apoptosis in human multiple myeloma cells. Blood 103, 2761–2770. 10.1182/blood-2003-09-3037 14645003

[B11] Debnath A. Calvet C. M. Jennings G. Zhou W. Aksenov A. Luth M. R.. (2017). CYP51 is an essential drug target for the treatment of primary amoebic meningoencephalitis (PAM). PLoS Negl. Trop. Dis. 11:e0006104. 10.1371/journal.pntd.0006104 29284029 PMC5746216

[B12] Debnath A. Tunac J. B. Galindo-Gómez S. Silva-Olivares A. Shibayama M. McKerrow J. H. (2012). Corifungin, a new drug lead against Naegleria, identified from a high-throughput screen. Antimicrob. Agents Chemother. 56, 5450–5457. 10.1128/AAC.00643-12 22869574 PMC3486592

[B13] De Jonckheere J. F. (2011). Origin and evolution of the worldwide distributed pathogenic amoeboflagellate *Naegleria fowleri*. Infect. Genet. Evol. 11, 1520–1528. 10.1016/j.meegid.2011.07.023 21843657

[B14] Duma R. J. Finley R. (1976). *In vitro* susceptibility of pathogenic Naegleria and Acanthamoeba speicies to a variety of therapeutic agents. Antimicrob. Agents Chemother. 10, 370–376. 10.1128/AAC.10.2.370 984777 PMC429749

[B15] Gautam P. L. Sharma S. Puri S. Kumar R. Midha V. Bansal R. (2012). A rare case of survival from primary amebic meningoencephalitis. Indian J. Crit. Care Med. 16, 34–36. 10.4103/0972-5229.94432 22557831 PMC3338237

[B16] Goswick S. M. Brenner G. M. (2003). Activities of azithromycin and amphotericin B against *Naegleria fowleri in vitro* and in a mouse model of primary amebic meningoencephalitis. Antimicrob. Agents Chemother. 47, 524–528. 10.1128/AAC.47.2.524-528.2003 12543653 PMC151771

[B17] Imai H. Masayasu H. Dewar D. Graham D. I. Macrae I. M. (2001). Ebselen protects both gray and white matter in a rodent model of focal cerebral ischemia. Stroke 32, 2149–2154. 10.1161/hs0901.095725 11546910

[B18] Jain R. Prabhakar S. Modi M. Bhatia R. Sehgal R. (2002). Naegleria meningitis: a rare survival. Neurol. India 50, 470–472. 12577098

[B19] Jayakumar A. R. Tong X. Y. Ruiz-Cordero R. Bregy A. Bethea J. R. Bramlett H. M.. (2014). Activation of NF-κB mediates astrocyte swelling and brain edema in traumatic brain injury. J. Neurotrauma 31, 1249–1257. 10.1089/neu.2013.3169 24471369 PMC4108982

[B20] Johnson R. O. Cope J. R. Moskowitz M. Kahler A. Hill V. Behrendt K.. (2016). Notes from the field: primary Amebic Meningoencephalitis associated with exposure to swimming pool water supplied by an Overland Pipe - Inyo County, California, (2015). MMWR Morb. Mortal. Wkly. Rep. 65:424. 10.15585/mmwr.mm6516a4 27123690

[B21] Juliana C. Fernandes-Alnemri T. Wu J. Datta P. Solorzano L. Yu J. W.. (2010). Anti-inflammatory compounds parthenolide and Bay 11-7082 are direct inhibitors of the inflammasome. J. Biol. Chem. 285, 9792–9802. 10.1074/jbc.M109.082305 20093358 PMC2843228

[B22] Kamigata N. Izuka H. Izuoka A. Kobayashi M. (1986). Photochemical reaction of 2-aryl-1,2-benzisoselenazol-3(2H)-ones. Bull. Chem. Soc. Jpn. 59, 2179–2183. 10.1246/bcsj.59.2179

[B23] Keller S. A. Hernandez-Hopkins D. Vider J. Ponomarev V. Hyjek E. Schattner E. J.. (2006). NF-kappaB is essential for the progression of KSHV- and EBV-infected lymphomas *in vivo*. Blood 107, 3295–3302. 10.1182/blood-2005-07-2730 16380446 PMC1432097

[B24] Koedel U. Bayerlein I. Paul R. Sporer B. Pfister H. W. (2000). Pharmacologic interference with NF-κB activation attenuates central nervous system complications in experimental Pneumococcal meningitis. J. Infect. Dis. 182, 1437–1445. 10.1086/315877 11023466

[B25] Lawande R. V. John I. Dobbs R. H. Egler L. J. (1979). A case of primary amebic meningoencephalitis in Zaria, Nigeria. Am. J. Clin. Pathol. 71, 591–594. 10.1093/ajcp/71.5.591 453078

[B26] Lee J. Kim J. H. Sohn H. J. Yang H. J. Na B. K. Chwae Y. J.. (2014). Novel cathepsin B and cathepsin B-like cysteine protease of *Naegleria fowleri* excretory-secretory proteins and their biochemical properties. Parasitol. Res. 113, 2765–2776. 10.1007/s00436-014-3936-3 24832815

[B27] Lee J. Rhee M. H. Kim E. Cho J. Y. (2012). BAY 11-7082 is a broad-spectrum inhibitor with anti-inflammatory activity against multiple targets. Mediators Inflamm. 2012:416036. 10.1155/2012/416036 22745523 PMC3382285

[B28] Loschiavo F. Ventura-Spagnolo T. Sessa E. Bramanti P. (1993). Acute primary meningoencephalitis from entamoeba *Naegleria Fowleri*. Report of a clinical case with a favourable outcome. Acta Neurol. 15, 333–340. 8304081

[B29] Lynch E. Kil J. (2009). Development of ebselen, a glutathione peroxidase mimic, for the prevention and treatment of noise-induced hearing loss. Semin. Hear. 30, 047–055. 10.1055/s-0028-1111106

[B30] McCurdy D. K. Frederic M. Elkinton J. R. (1968). Renal tubular acidosis due to amphotericin B. N. Engl. J. Med. 278, 124–130. 10.1056/NEJM196801182780302 5634966

[B31] Nau R. Sörgel F. Eiffert H. (2010). Penetration of drugs through the blood-cerebrospinal fluid/blood-brain barrier for treatment of central nervous system infections. Clin. Microbiol. Rev. 23, 858–883. 10.1128/CMR.00007-10 20930076 PMC2952976

[B32] Nikawa T. Schuch G. Wagner G. Sies H. (1994). Interaction of ebselen with glutathione S-transferase and papain *in vitro*. Biochem. Pharmacol. 47, 1007–1012. 10.1016/0006-2952(94)90411-1 8147899

[B33] Parnham M. Sies H. (2000). Ebselen: prospective therapy for cerebral ischaemia. Expert Opin. Investig. Drugs 9, 607–619. 10.1517/13543784.9.3.607 11060699

[B34] Poungvarin N. Jariya P. (1991). The fifth nonlethal case of primary amoebic meningoencephalitis. J. Med. Assoc. Thai. 74, 112–115. 2056258

[B35] Proffitt R. T. Satorius A. Chiang S. M. Sullivan L. Adler-Moore J. P. (1991). Pharmacology and toxicology of a liposomal formulation of amphotericin B (AmBisome) in rodents. J. Antimicrob. Chemother. 28(Suppl. B.), 49–61. 10.1093/jac/28.suppl_B.49 1778892

[B36] Rajguru R. (2013). Military aircrew and noise-induced hearing loss: prevention and management. Aviat. Space Environ. Med. 84, 1268–1276. 10.3357/ASEM.3503.2013 24459798

[B37] Renslo A. R. McKerrow J. H. (2006). Drug discovery and development for neglected parasitic diseases. Nat. Chem. Biol. 2, 701–710. 10.1038/nchembio837 17108988

[B38] Saito I. Asano T. Sano K. Takakura K. Abe H. Yoshimoto T.. (1998). Neuroprotective effect of an antioxidant, ebselen, in patients with delayed neurological deficits after aneurysmal subarachnoid hemorrhage. Neurosurgery 42, 269–277. discussion: 277–268. 10.1097/00006123-199802000-00038 9482177

[B39] Salom J. B. Perez-Asensio F. J. Burguete M. C. Marin N. Pitarch C. Torregrosa G. (2004). Single-dose ebselen does not afford sustained neuroprotection to rats subjected to severe focal cerebral ischemia. Eur. J. Pharmacol. 495, 55–62. 10.1016/j.ejphar.2004.05.024 15219820

[B40] Scheidt K. A. Roush W. R. McKerrow J. H. Selzer P. M. Hansell E. Rosenthal P. J. (1998). Structure-based design, synthesis and evaluation of conformationally constrained cysteine protease inhibitors. Bioorg. Med. Chem. 6, 2477–2494. 10.1016/S0968-0896(98)80022-9 9925304

[B41] Schuster F. L. Visvesvara G. S. (2004). Opportunistic amoebae: challenges in prophylaxis and treatment. Drug Resist. Updat. 7, 41–51. 10.1016/j.drup.2004.01.002 15072770

[B42] Seidel J. S. Harmatz P. Visvesvara G. S. Cohen A. Edwards J. Turner J. (1982). Successful treatment of primary amebic meningoencephalitis. N. Engl. J. Med. 306, 346–348. 10.1056/NEJM198202113060607 7054710

[B43] Selvakumar K. Shah P. Singh H. B. Butcher R. J. (2011). Synthesis, structure, and glutathione peroxidase-like activity of amino acid containing ebselen analogues and diaryl diselenides. Chemistry 17, 12741–12755. 10.1002/chem.201100930 21956838

[B44] Shakoor S. Beg M. A. Mahmood S. F. Bandea R. Sriram R. Noman F.. (2011). Primary amebic meningoencephalitis caused by *Naegleria fowleri*, Karachi, Pakistan. Emerging Infect. Dis. 17, 258–261. 10.3201/eid1702.100442 21291600 PMC3204751

[B45] Singh S. N. Patwari A. K. Dutta R. Taneja N. Anand V. K. (1998). Naegleria meningitis. Indian Pediatr. 35, 1012–1015. 10216726

[B46] Vargas-Zepeda J. Gómez-Alcalá A. V. Vásquez-Morales J. A. Licea-Amaya L. De Jonckheere J. F. Lares-Villa F. (2005). Successful treatment of *Naegleria fowleri* meningoencephalitis by using intravenous amphotericin B, fluconazole and rifampicin. Arch. Med. Res. 36, 83–86. 10.1016/j.arcmed.2004.11.003 15900627

[B47] Visvesvara G. S. (2010). Amebic meningoencephalitides and keratitis: challenges in diagnosis and treatment. Curr. Opin. Infect. Dis. 23, 590–594. 10.1097/QCO.0b013e32833ed78b 20802332

[B48] Visvesvara G. S. Moura H. Schuster F. L. (2007). Pathogenic and opportunistic free-living amoebae: acanthamoeba spp., *Balamuthia mandrillaris, Naegleria fowleri*, and *Sappinia diploidea*. FEMS Immunol. Med. Microbiol. 50, 1–26. 10.1111/j.1574-695X.2007.00232.x 17428307

[B49] Visvesvara G. S. Stehr-Green J. K. (1990). Epidemiology of free-living ameba infections. J. Protozool. 37, 25S−33S. 10.1111/j.1550-7408.1990.tb01142.x 2258827

[B50] Vyas I. K. Jamerson M. Cabral G. A. Marciano-Cabral F. (2015). Identification of peptidases in highly pathogenic vs. weakly pathogenic *Naegleria fowleri* amebae. J. Eukaryot. Microbiol. 62, 51–59. 10.1111/jeu.12152 25066578

[B51] Wang A. Kay R. Poon W. S. Ng H. K. (1993). Successful treatment of amoebic meningoencephalitis in a Chinese living in Hong Kong. Clin. Neurol. Neurosurg. 95, 249–252. 10.1016/0303-8467(93)90132-Z 8242970

[B52] Yamaguchi T. Sano K. Takakura K. Saito I. Shinohara Y. Asano T.. (1998). Ebselen in acute ischemic stroke: a placebo-controlled, double-blind clinical trial. Ebselen Study Group. Stroke 29, 12–17. 10.1161/01.STR.29.1.12 9445321

[B53] Yoder J. S. Eddy B. A. Visvesvara G. S. Capewell L. Beach M. J. (2010). The epidemiology of primary amoebic meningoencephalitis in the USA, 1962-2008. Epidemiol. Infect. 138, 968–975. 10.1017/S0950268809991014 19845995

[B54] Yoder J. S. Straif-Bourgeois S. Roy S. L. Moore T. A. Visvesvara G. S. Ratard R. C.. (2012). Primary amebic meningoencephalitis deaths associated with sinus irrigation using contaminated tap water. Clin. Infect. Dis. 55, e79–e85. 10.1093/cid/cis626 22919000 PMC11307261

[B55] Zanotto-Filho A. Delgado-Cañedo A. Schröder R. Becker M. Klamt F. Moreira J. C. (2010). The pharmacological NFkappaB inhibitors BAY117082 and MG132 induce cell arrest and apoptosis in leukemia cells through ROS-mitochondria pathway activation. Cancer Lett. 288, 192–203. 10.1016/j.canlet.2009.06.038 19646807

